# A retrospective study of Cerebrolysin in patients with moderate to severe traumatic brain injury: Cognitive and functional outcomes

**DOI:** 10.25122/jml-2023-0125

**Published:** 2023-07

**Authors:** Claudio Soto, Pablo Salinas, Daniel Muñoz, Sandra Olivares, Javiera González, Virginia Sáez, Violeta Romero

**Affiliations:** 1 Departament of Research and Development , Clinica Los Coihues, Mutual de Seguridad C.Ch.C, Santiago, Chile; 2 Departament of Neurorehabilitation, Mutual de Seguridad C.Ch.C., Universidad de Chile, Santiago, Chile; 3 Departament of Biostatistics, School of Public Health, Universidad de Chile, Santiago, Chile; 4 Departament of Research and Development, Clinica Los Coihues, Santiago, Chile

**Keywords:** brain injuries, TBI, Cerebrolysin, patient outcome assessment, rehabilitation, neurological rehabilitation, TBI: Traumatic brain injury, NMDAR: N-methyl-D-aspartate receptors, CNS: Central Nervous System, NF: Neurotrophic Factors, HCMS: Hospital Clínico de la Mutual de Seguridad, GCS: Glasgow Coma Scale, EEG: ElectroEncephaloGram, RLAS: Rancho Los Amigos Scale, DRS: Disability Rating Scale, FIM: Functional Independence Measure, MoCA: Montreal Cognitive Assessment, LOTCA: Lowenstein Occupational Therapy Cognitive Assessment, FAB: Frontal Assessment Battery

## Abstract

In this retrospective study, we aimed to evaluate the effects of the neurotrophic compound Cerebrolysin on executive, cognitive, and functional performance in patients with traumatic brain injury (TBI) with a highly severe disability level. A total of 44 patients were included in the study, with 33 patients in the control group and 11 patients in the interventional group who received intravenous infusions of 30 mL Cerebrolysin. Both groups received standard rehabilitation therapy following the rehabilitation protocol for patients with TBI at Hospital Clínico Mutual de Seguridad. Functional and cognitive scales were evaluated at baseline, at four months, and at the endpoint of the intervention therapy at seven months (on average). The results revealed a significant improvement in the Cerebrolysin-treated group compared to the control group. Specifically, patients who received Cerebrolysin showed a moderate residual disability and a significant reduction in the need for care. Concerning the promising results and considering the limitations of the retrospective study design, we suggest that randomized controlled studies be initiated to corroborate the positive findings for Cerebrolysin in patients with moderate to severe brain trauma.

## INTRODUCTION

Traumatic brain injury (TBI) is defined as the physical injury and the functional deterioration of the cranial contents due to a sudden exchange of mechanical energy between the encephalic-cranial set and a traumatic agent [1]. TBI has emerged as a critical public health and socio-economic concern [2], becoming a leading cause of death and permanent disability, especially among young adults. The World Health Organization (WHO) had estimated that by 2020 it would be the leading cause of death and disability in the world, with an estimated 10 million people affected annually [3].

TBI contributes to worldwide death and disability more than any other traumatic insult. An estimated 1269 million individuals will suffer a TBI each year, the vast majority of which will be mild (81%) and moderate (11%) in severity [4]. In Chile, TBI is the primary cause of death in individuals between 20 and 40 years of age, and it is also a significant cause of neurological sequelae in patients in the working-age population [1]. Early rehabilitation has been shown to contribute to better outcomes, including shorter rehabilitation duration, improved functional capacity, and increased employment rates [5].

The pathophysiology of TBI involves complex processes such as neuroprotection, neurorecovery, and neurotoxicity, which share common pathways, including the activity of N-methyl-D-aspartate receptors (NMDAR) [6, 7]. Neuromodulation is the optimization of the typical biological process that can potentially either produce cellular death or neuroregeneration. This process involves multimodal rehabilitation through exogenous neurotrophic factors that may produce immediate neuroprotection, paired with an effect on the long-term repair processes, like endogenous regulation. It has been determined in experimental studies that a stimulating drug of the central nervous system (CNS) is potentially beneficial in recovery and requires a stimulation-rich environment [8].

Neurotrophic factors (NF) are polypeptides, naturally synthesized by all types of cells in CNS and other tissues. Their activity is essential for the development and functional maintenance of the CNS since they stimulate proliferation and cell differentiation, axonal and dendritic growth. In addition, NF stimulates neuronal plasticity and synaptic activity, which is essential for the CNS's ability to reorganize itself spontaneously after different injuries and in the learning processes [9].

Cerebrolysin (Renacenz, EVER Neuro Pharma, Austria) is the only available drug that contains active fragments of different neurotrophic factors of low molecular weight that can cross the hematoencephalic barrier. These peptides mimic the action of endogenous neurotrophic factors and exert an immediate pleiotropic neuroprotective effect and long-term activity in brain recovery [10]. The effect of Cerebrolysin has been analyzed in several clinical studies of TBI. Wong *et al*. conducted a study involving 21 TBI patients who received Cerebrolysin and compared them with a non-concurrent control group [11]. Although the difference was not statistically significant, 67% of the Cerebrolysin-treated patients showed a better outcome compared to the control group. Alvarez *et al*. [12] studied 39 patients with TBI, with 20 receiving Cerebrolysin and 19 undergoing the usual neurorehabilitation program with a 21-month follow-up. The study found that the deceleration of EEG activity was significantly reduced in TBI patients treated with Cerebroylsin within the first month of treatment and, after three months, correlated with improved attention and working memory.

Onose *et al*. [8] conducted a comparative study among 69 patients with TBI treated with Cerebrolysin and 70 controls. The conclusion was that the administration of Cerebrolysin statistically significantly accelerates neurorecovery and improves neurorehabilitation outcomes. Similarly, Poon *et al*. [13] observed that using Cerebrolysin after TBI positively affects functional scales. Finally, Muresanu *et al*. [14] confirmed the positive impact of the multimodal, biological agent Cerebrolysin for overall outcome after moderate to severe TBI, as measured by a multidimensional approach.

Our study aimed to evaluate the effect of Cerebrolysin treatment in patients with moderate to severe TBI as part of neurorehabilitation.

## MATERIAL AND METHODS

### Study design

This retrospective study compared patients with traumatic brain injury (TBI) who received Cerebrolysin treatment (intervention group) with a control group of patients with TBI who did not receive the intervention. The study was conducted at the Instituto de Rehabilitación del Hospital Clínico de la Mutual de Seguridad (HCMS) between June 2010 and June 2012.

### Treatment protocol

Both groups received standard rehabilitation therapy following the protocol for patients with TBI at HCMS. This included pharmacological treatment according to medical indications and rehabilitation therapy provided by physical therapists, occupational therapists, and speech and language pathologists. The therapy was tailored to the functional level of each patient and adapted as they progressed. The treatment group received intravenous infusions of 30 ml/day of Cerebrolysin for ten consecutive days once per month for three consecutive months.

### Inclusion criteria

1. Clinical diagnosis of TBI and a Glasgow Coma Scale (GCS) score of 5–13 at hospital admission.
2. Isolated TBI
3. Age between 18 and 80 years.

**Exclusion criteria**

1. Patients with polytrauma
2. Patients with spinal cord injury.
3. History of intracranial interventions as well as ischemic or hemorrhagic stroke.
4. Evidence of pre-existing primary health conditions, such as cancer, hematological, renal, hepatic, or coronary disease, and psychiatric disorders.
5. Dementia.
6. Any neurological or non-neurological condition independent from TBI that might influence the functional outcome or other efficacy outcome measures.
7. Patients with penetrating brain injury.

### Outcome Measures

Cognitive and functional assessments were performed at baseline, after which the treatment group received Cerebrolysin for ten consecutive days once a month for three consecutive months. The first assessment was conducted at the beginning of Month 4, and the second was conducted during Month 7 (final assessment). The following scales were used: the Rancho Los Amigos Scale (RLAS, numerical scale from 1 to 10) [15], the Disability Rating Scale (DRS, numerical scale from 1 to 30) [16], and the Functional Independence Measure (FIM, numerical scale from 0 to 35 in the cognitive domain; 0 to 91 in the motor domain; and 0 to 126 in the total) [17]. In addition, the following assessments were performed at baseline and the final evaluation: Montreal Cognitive Assessment (MoCA, numerical scale from 1 to 30) [18], Lowenstein Occupational Therapy Cognitive Assessment (LOTCA percentile scale from 1 to 100) [19) and Frontal Assessment Battery (FAB, numerical scale from 1 to 18) [20]. The rehabilitation follow-up time was established from the date of the accident.

### Statistical Analysis

Numeric variables were described using the mean and standard deviation or median and interquartile range and compared between groups using t-tests or a non-parametric test, as appropriate. Categorical variables were described using frequencies and percentages and compared between groups using the Chi-square distribution or Fischer’s exact test. RLAS, DRS, and MoCA scores were described using the median and interquartile range, and group comparisons were made using the Wilcoxon-Mann-Whitney test. For all other scales, the scores were described using mean and standard deviation, and groups were compared using t-tests. The timely evolution of clinical scale scores was analyzed using a Generalized Estimating Equations (GEE) model. A two-sided p-value lower than 0.05 was considered statistically significant. Data were tabulated in Microsoft Excel 2010 and analyzed in Stata^®^ 13.1 (StataCorp, College Station, TX).

## RESULTS

The study included 11 patients (25%) in the active treatment group who received Cerebrolysin and 33 matched patients (75%) in the control group who did not receive the drug. The evolution of clinical evaluation scale scores over time was compared between the two groups.

Table 1 shows the baseline characteristics, and both groups were comparable in age, GCS, and type of injury, as well as in the cognitive and functional scales at admission.

**Table 1:** Baseline characteristics of study patients

	Cerebrolysin (n=11)	Control group (n=33)	p-value
Age (years)	47.54 (2.35)	48.63 (6.08)	0.8395
GCS	8 (5-13)	12 (7- 13)	0.4097
Injury type			
Extradural	3/11 (27.27%)	10/33 (30.30%)	0.8353
Hemorrhage			
Subdural	5/11 (45.45%)	15/33 (45.45%)	1.000
Hemorrhage			
Subarachnoid hemorrhage	2/11 (18.18%)	5/33 (15.15%)	0.8130
Skull base fracture	1/11 (9.09%)	3/33 (9.09%)	1.000
RLAS	3 (2-4)	3 (3-4)	0.4273
DRS	20 (15-22)	21 (18-23)	0.6051
MOCA	7 (0-18)	9 (6-12.5)	0.8406
LOTCA	47.13 (33.20)	38.50 (30.20)	0.6448
FAB	5.53 (3.62)	6.00 (3.00)	0.8380
FIM (Total)	32,16 (25.67)	34.55 (34.77)	0.8094
FIM (Cognitive)	9.28 (5.79)	9.73 (9.13)	0.8512
FIM (Movement)	22,91 (21.96)	24.82 (26.04)	0.8134

The GEE analysis demonstrated a significant interaction between time and outcome for each scale, indicating that both groups significantly improved in the cognitive and functional domains over time. Likewise, the GEE analysis revealed a significant treatment effect of Cerebrolysin for each scale, indicating that the Cerebrolysin-treated patients had a significantly greater improvement in functional and cognitive outcomes compared to the control patients (Table 2).

**Table 2:** GEE analysis

Variables	Time ß (95%Cl); p-value	Cerebrolysin ß (95%Cl); p-value
RLAS	1.23 (.09;1.51); p=0.001 **	2.40 (1.69-3.11); p=0.001 **
DRS	-8.11 (-10.62;-5.15); p=0.001 **	-3.78 (-4.77; -2.79) p<0.0001**
MOCA	10.08 (6.25; 13.91) p<0.0001 **	11.35 (7.05; 13.95) p<0.0001 **
LOTCA	31.94 (19.47; 44.42) p<0.0001	1.48 (.45; 1.56); p=0.003 **
FAB	7.33 (5.11; 9.56) p<0.0001 **	5.08 (3.25; 9.93) p<0.0001 **
FIM (Total)	17.04 (12.00;22.08); p<0.0001 **	18.52 (6.91; 26.14); p= 0.004 **
FIM (Cognitive)	5.00 (3.66;6.35) p<0.0001 **	6.11 (2.51; 9.72); p=0.001 **
FIM (Movement)	12.69 (8.71;16.67); p<0.0001 **	15.37 (3.84; 26.91); p=0.009 **

** p < 0,01

**Figure 1:**
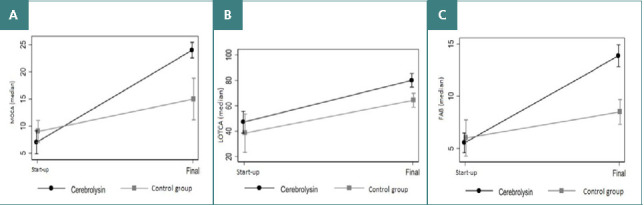
Cognitive outcomes at the end of the observation period

In the cognitive domain, the results for the MoCA, LOTCA, and FAB showed that while baseline scores at the beginning of the study were comparable between groups, significant group differences became evident at the end of the observation period, indicating a significant treatment effect in favor of Cerebrolysin (Figure 1 a-c).

**Figure 2:**
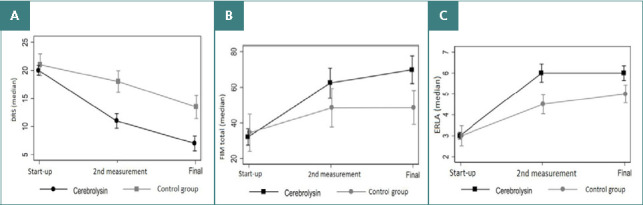
Functional outcomes at the end of the observation period

Similarly, a very similar picture was observed for the functional outcome scales (Figure 2 a-c) with significant group differences in favor of the Cerebroylsin-treated patients in the DRS, RLAS, FIM (cognitive, motor, and total) scales at the study endpoint, again indicating a significant treatment effect of Cerebroylsin in patients with severe TBI.

## DISCUSSION

The control and treatment groups were comparable in age, GCS, type of lesion, and evaluation scales used, ensuring the comparability of the two groups.

Our study demonstrated a consistent and significant improvement in neurorehabilitation outcomes for patients who received Cerebrolysin compared to the control group. This treatment effect was observed across multiple domains of neurological deficits following TBI.

Effects in the cognitive domain were most pronounced in the MoCA scale. Even though patients on Cerebrolysin started with lower scores at baseline, they achieved significantly higher final scores and better outcomes than those of the control group. Notably, patients on Cerebrolysin even surpassed, on average, the cut-off for typical global cognitive performance (24 points), including executive visual-spatial, categorization, memory, attention, language, and orientation performance. In contrast, while control patients showed a slight cognitive improvement over time, they also failed to reach cognitive scores beyond the cut-off. This is confirmed by similar results in the cognitive-perceptual evaluation using the LOTCA score, where the treatment group showed an approximately 20% higher improvement compared to the control group.

Regarding the global disability evaluation using the DRS, both groups started with an extremely severe disability level. While the treatment group reached a moderate disability level at the study endpoint, the control group, despite some positive effects over time, only improved to a severe disability degree at the final evaluation.

On the RLAS scale, both groups started with response levels indicating the need for complete care assistance. However, at the end of the study, the control group showed an inappropriately confused response level, with the need for maximum assistance, compared to the treatment group, which showed an appropriately confused response with only moderate assistance requirements.

In terms of functional outcome, as evidenced by the FIM (total) scale, patients in the control group remained stable in their functional evolution up until the study endpoint, whereas patients on Cerebrolysin treatment showed a continuous improvement with a significantly better functional outcome at the end of the study.

Overall, the findings of our study are very robust, demonstrating a significant superiority of Cerebrolysin over standard rehabilitation alone in various clinical domains. Furthermore, we observed that the administration of Cerebrolysin in the sample of TBI patients reduced the need for assistance provided by the teams and caregivers during the neurorehabilitation period.

Based on the positive findings of this study, further exploration in this field should involve randomized clinical trials to confirm the observed results. Importantly, Cerebrolysin treatment may significantly impact the improvement of clinical outcomes in terms of global executive, cognitive, and functional performance in patients for which only limited treatment options exist to date.

The study confirms the advantages of Cerebrolysin in cases of moderate to severe TBI, reinforcing the rationale for employing multimodal agents and the multidimensional approach in clinical research [14].

An evident strength of the current study was the homogenous patient sample enrolled despite the considerable heterogeneity of TBI patients, allowing for an unbiased interpretation of the study results. At the same time, the retrospective design and the relatively low number of patients are considered study limitations.

## CONCLUSION

This data analysis outlines the advantages in functional outcomes observed among patients who underwent treatment with Cerebrolysin following cases of moderate to severe TBI. This information may provide valuable information for investigators, so further studies that seek to improve clinical trial designs are suggested.
